# The Enteric Microbiome in Early-Onset Colorectal Cancer: A Comprehensive Review of Its Role as a Biomarker of Disease

**DOI:** 10.14309/ctg.0000000000000864

**Published:** 2025-05-28

**Authors:** Jesús M. Luévano, Julia Liu, Thaddeus Stappenbeck

**Affiliations:** 1 Division of Gastroenterology, Department of Medicine, Morehouse School of Medicine, Atlanta, Georgia, USA;; 2 Department of Inflammation and Immunity, Learner Research Institute, Cleveland Clinic College of Medicine, Cleveland, Ohio, USA.

**Keywords:** microbiome, early-onset colorectal cancer, biomarker, review

## Abstract

Early-onset colorectal cancer (EoCRC), a distinct entity from late-onset colorectal cancer (LoCRC), continues to increase in incidence. Known risk factors for LoCRC have been explored to explain this trend, but do not account for it completely. The gastrointestinal microbiome has been associated with LoCRC and additional risk factors of disease; however, it is only now being investigated in the context of EoCRC. A better understanding of the microbiome's function in EoCRC could elucidate its role in the increasing incidence of EoCRC. This article reviews the state of literature related to studies specifically isolating microbiome-related changes in EoCRC compared with LoCRC and age-matched controls. Several studies reviewed in this article highlight the varied results of overall diversity and specific bacteria that are influenced by EoCRC, and the utility of these unique changes to predict for EoCRC. Although the microbiome can be useful in understanding EoCRC, to better predict for disease the microbiome must be studied in more diverse populations and with deeper, more functional characterization in a manner that allows for transference of findings among future studies. These studies indicate that the enteric microbiome holds significant potential as a biomarker for disease but has yet to fully meet an understanding necessary for direct clinical utilization.

## Graphical abstract

**Figure:**
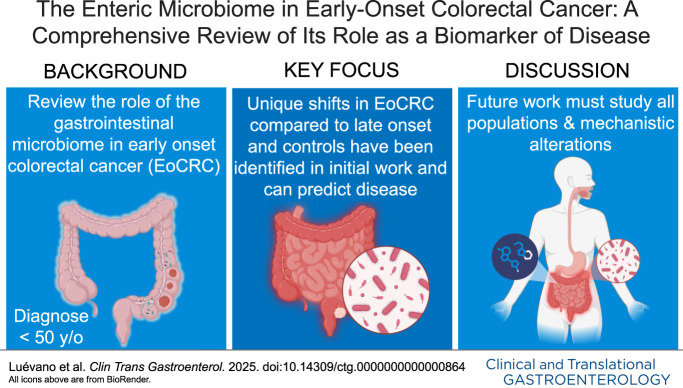

## RISE OF EARLY-ONSET COLORECTAL CANCER

Colorectal cancer (CRC) ranks third in cancer mortality in the United States (1). Incidence increases with age, doubling in each successive 5-year group from ages 20 to 60 years (2). Improvements in mortality stem from universal screening, advanced surgical options, and newer adjuvant chemotherapies (3). However, early-onset colorectal cancer (EoCRC), defined as CRC before age 50 years, is rising in the United States, Europe, and Asia, accounting for 13% of all CRC, up from 6% 2 decades prior (1,4–9). Following updated specialty organizations recommendations (10–12), in 2021, the US Preventive Services Task Force reduced the universal screening age to 45 years (13).

Most EoCRC cases seem sporadic, independent of genetic syndromes, with ∼70%–80% arising from somatic mutations (4,14,15). Compared with traditional CRC, dubbed average-onset CRC or late-onset CRC (LoCRC), EoCRC generally is more aggressive, diagnosed at advanced stages, and with greater metastatic potential (16,17). EoCRC incidence and mortality in Non-Hispanic Black-Americans is nearly double that of White patients (18–20). Demographic factors associated with CRC also associate with EoCRC, including obesity and sedentary lifestyle (21,22), diabetes (23), and unhealthy diets (24,25). Of these, no singular factor provides a compelling explanation for the trend in EoCRC (7), with some studies demonstrating null results (26). Over the past 50 years, EoCRC incidence initially decreased from 1975 to 1990, but has since increased with differing magnitudes across 5-year age groups, suggestive of birth cohort effects in those born after 1960, possibly from early-life exposures (5,6,12,27).

Diet has well-demonstrated CRC associations, particularly Western diets low in fiber and rich in red meat (26,28,29) (vs prudent diets (30)), alcohol (24,26,28,31), and sugary drinks (32). Studies demonstrate stronger associations with Western diets and early-onset advanced adenomas in the distal colon/rectum (where EoCRC is more common) compared with the proximal colon (24), with high-fat avoidance being protective (25). Sugar-sweetened drinks associated with increased EoCRC risk (33), particularly in women (34), although fructose associated with increased proximal but not distal CRC mortality (35). Heavy alcohol use was associated with higher rates of EoCRC (36–38), although 1 study found increased risk with abstinence and heavy use (39).

As further research links CRC risk factors to EoCRC, an important mechanism intertwined with metabolic syndromes, alcohol use, and dietary changes is the gastrointestinal microbiome (40–47).

## UPPER AND LOWER GASTROINTESTINAL MICROBIOME IN CRC

The human microbiome includes the totality of genomes encoding the transcriptional, translational, and metabolic functions of our commensal microbes. CRC-associated microbiome alterations, including decreased community diversity and shifts at higher-order taxonomic levels (e.g., Bacteroides and Firmicutes), have been sought for diagnostic, prophylactic, and therapeutic applications (40–47). Patients with CRC had higher levels of *Fusobacterium* (42,45–48), particularly mucosal samples (43), which produces the short-chain fatty acid (SCFA) butyrate, the primary colonocyte energy source and modulation of which is associated with CRC carcinogenesis (49–51). *Apc*^*Min/+*^ mice exposed to *F. nucleatum* demonstrated increased tumor multiplicity and tumor-infiltrating myeloid cells, which can promote tumor progression (46). Interestingly, Enterobacteriaceae, which can produce DNA-damaging genotoxins (52), was decreased in CRC (45).

Common fecal bacteria associated with CRC carcinogenesis include *Bacteroides fragilis* (47,53), *Escherichia coli* (54), *Enterococcus faecalis* (55), *Streptococcus gallolyticus* (56), and *Morganella morganii* (57). The microbiome as a CRC biomarker has been demonstrated (58–66). Several prevalent oral bacteria, *Peptostreptococcus* (43) and *F. nucleatum* (67–69), have been associated with CRC, and unique oral microbiome shifts were successfully used to develop predictive models (60,62). Chen et al, developed predictive models for adenomas and CRC (area under the curve [AUC] = 0.84 and 0.93) using bacterial community and serum metabolomic data (59). Noninvasive screening alternatives featuring microbiome and metabolic data can improve upon accuracy of older serologic markers, cell-free DNA (70–72), and even fecal-based immunochemical tests (73,74), a means to increase access for underserved populations (18,75–77). However, few studies evaluate the role of the microbiome in EoCRC vs age matched controls or LoCRC.

## MICROBIOME IN EoCRC

Sixteen studies were identified through searches on PubMed and Google Scholar using search items: “early onset colorectal cancer,” “EoCRC,” “microbiome,” “microbiota,” “16S,” “metagenomics,” “sequencing,” “metabolome,” and “young onset colorectal cancer.” Eleven were from the United States (78–88) and 5 China (40,89–92). All featured comparisons with EoCRC, varying in methodology for sample collection, sequencing, statistical analyses, and comparator group (LoCRC or age-matched controls). Prior studies primarily featured LoCRC or lacked age stratification (40–48,58–66). Only a few studies (40,81,83–86,88–91) and abstracts (78–80,82,87) specifically evaluate microbiome alterations in EoCRC (Table 1).

**Table 1.** Study design and patient metadata in early-onset colorectal cancer microbiome studies

Study	Year	Total subjects	LoCRC cases (median age)	EoCRC cases (median age)	Controls (median age)	Race: % (n) White/Black	Sample types (sequencing methods)	Study location	PMID/DOI
Keshinro et al^a,b^	2020	275	114 (70)^c^	24 (33.6)^c^	137	Not stated	Tumor tissue (NGS panel)	New York, NY, USA	10.1200/jco.2020.38.15_suppl.e16070
Jin et al^a^	2022	358	304	54	0	Not stated	Tumor tissue (RNA-Seq)	Columbus, OH, USA	10.1016/j.annonc.2022.04.426
Kharofa et al	2022	1,301	611	81	609 (60)	Not stated	Stool (metagenome), meta-analysis of 11 studies	Cincinnati, OH, USA	36056757
Dave et al^a^	2023	227	Not stated	Not stated	0	Not stated	Tumor tissue (16S)	Cleveland, OH, USA	10.1200/jco.2023.41.4_suppl.13
Weinberg et al	2023	63	27 (72)	36 (38)	0	Not stated	Tumor tissue (16S)	Washington DC, USA	10.1200/jco.2023.41.16_suppl.3530
White et al	2023	107	70 (61)	37 (42)	0	71%/7.5%	Tumor tissue and adjacent mucosa (metagenome)	Houston, TX, USA	37465976
Adnan et al	2024	1,393	619	82	Age matched: EoCRC controls 125/LoCRC controls 568	Not stated	Stool and mucosal tissue (16S)	Chicago, IL, USA	37967575
Barot et al	2024	276	140 (43)	136 (73)	0	83.6% (117)/14.3% (20)	Tumor tissue and adjacent mucosa (16S)	Cleveland Clinic, OH, USA	38306898
Hong et al^a^	2024	377	0	377	0	Not stated	Stool and mucosal tissue (16S, WGS, metabolomics)	St. Louis, MO, USA	10.1200/jco.2024.42.16_suppl.e15646
Jayakrishnan et al	2024	64	44	20	0	100%/0%	Tumor tissue (16S), serum (metabolomics)	Cleveland Clinic, OH, USA	39020083
Jin et al^a^	2024	Not stated	Not stated	Not stated	0	Not stated	Tumor tissue (RNA-Seq)	Columbus, OH, USA	10.1200/jco.2024.42.16_suppl.3580
Yang et al	2021	1,038	379 (64)	185 (40)	Age matched: EoCRC 217 controls (40)/LoCRC controls 257 (63)	Not stated	All stool (16S); stool subset of 200 samples (metagenome)	Shanghai, China	34799562
Xiong et al	2022	98	43	24	31	Not stated	Stool (16S)	Harbin, China	36389150
Xu et al	2022	39	19	20	0	Not stated	Tumor tissue (16S)	Shanghai, China	36119074
Kong et al	2023	434	130 (62)	114 (40)	Age matched: EoCRC controls 100 (39)/LoCRC controls 97 (62)	Not stated	Stool (metagenome/metabolomics)	Shanghai, China	35953094
Qin et al	2024	460	293	167	0	Not stated	Stool (metagenome), data from Yang et al and 8 public cohorts	Guangzhou, China	38649355

Studies were separated based on country of origin (United States or China). Total subjects (by case status [late onset vs early onset] and controls) are presented, as well as median age for that group^c^ (except for Keshinro et al, which provided mean age). Early-onset cases were defined as cases diagnosed before age 50, except for Kenshinro et al, that defined them as diagnosed before age 40. Late-onset cases are defined as diagnosed after age 50. When available, racial demographics were included.

16S, 16S rRNA sequencing; EoCRC, early-onset colorectal cancer; LoCRC, late-onset colorectal cancer; NGS, next-generation sequencing; RNA-Seq, RNA sequencing; WGS, whole genome sequencing.

a Abstracts.

b Defined EoCRC as younger than 40 years.

c Mean age.

### Differences in EoCRC vs LoCRC

**Figure 1.**
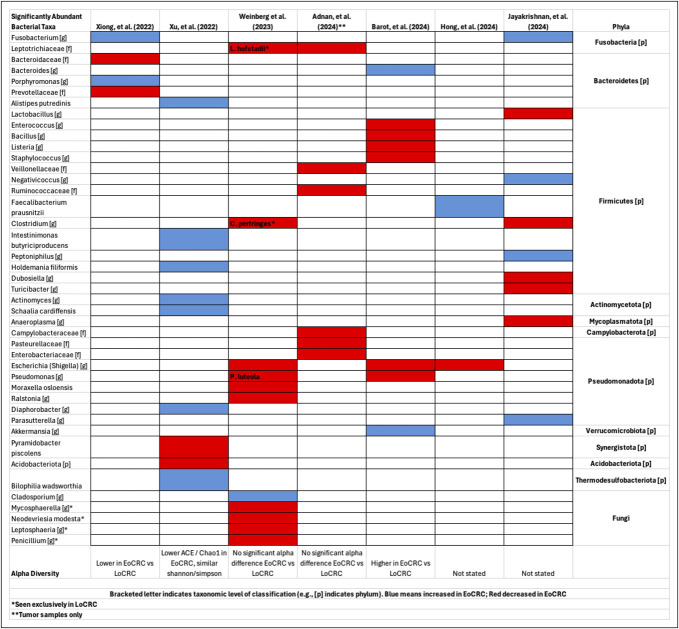
Enteric microbiota alterations between EoCRC and LoCRC. Reported alterations of the enteric microbiota that featured comparisons of EoCRC vs LoCRC were summarized. Blue and red boxes, respectively, indicate reported increases and decreases in relative abundance associated with EoCRC. Bracketed letter indicates taxonomic level of classification (e.g., [p] indicates phylum). When available, species data are included within the genus taxa box. A summary of alpha diversity findings is included at the bottom of the table for each study. Bacterial taxa were grouped based on similar phyla or kingdom for fungi. One species, *L. hofstadii*, was seen only in patients with LoCRC. The samples from Adnan et al, comprised tumor-based samples only. EoCRC, early-onset colorectal cancer; LoCRC, late-onset colorectal cancer.

Several microbial differences were consistent between EoCRC and LoCRC. Within the phyla Fusobacteria, EoCRC was associated with increased abundance of *Fusobacterium* (40,88) and decreased *Clostridium* (83,88) and the family Leptotrichiaceae (83,85) (Figure 1).

Greater proportions of *Fusobacterium*, associated with LoCRC in humans (42,45–48,67–69) and carcinogenesis in mice (43,46), provides a potential explanation for earlier and more aggressive disease in EoCRC. *Clostridium* decreased in CRC was increased after curative surgery, with alterations of carcinogenesis-associated deoxycholate bile acid (BA) producing function through *bai* operon, a microbiome-mediated functional change (93,94). *C. septicum* infections have been associated with CRC (95), and it grows well in the cecum (the most acidic colon segment (96)), whereas EoCRC more often presents distally, which may be related to lower proportions of *Clostridium*.

Within the phyla Pseudomonadota, decreases in *Eschericia* (83,86,87) and *Pseudomonas* (83,86) associated with EoCRC (Figure 1). *Pseudomonas* species can produce a biosurfactant with antitumorigenic properties against colon cancer cells (97), specifically the peptide Azurin-p28 that can enhance the effect of 5-fluorouracil (98). Loss of this capability may select for procarcinogenic states seen in EoCRC and explain why certain patients have greater 5-fluorouracil complications (99).

Remaining significant microbial taxa were limited to individual studies (Figure 1). There were several consistent bacterial associations with any CRC. *Clostridium symbiosum* was elevated in LoCRC and EoCRC compared with controls, as well as *Peptostreptococcus stomatis*, *Parvimonas micra*, and *Hungatella hathewayi* (92). This suggests predictive tools developed for LoCRC using microbial data may be able to successfully classify EoCRC. Alpha diversity variations in EoCRC, a measure of intraindividual microbial diversity, were not consistent, making it difficult to highlight a predictive signal.

Addressing the absence of global community shifts as a discriminatory signal will require deeper levels of microbial community profiling, larger training datasets, inclusion of other sampling sites, and functional profiling for better characterization.

### Differences in EoCRC vs age-matched controls

**Figure 2.**
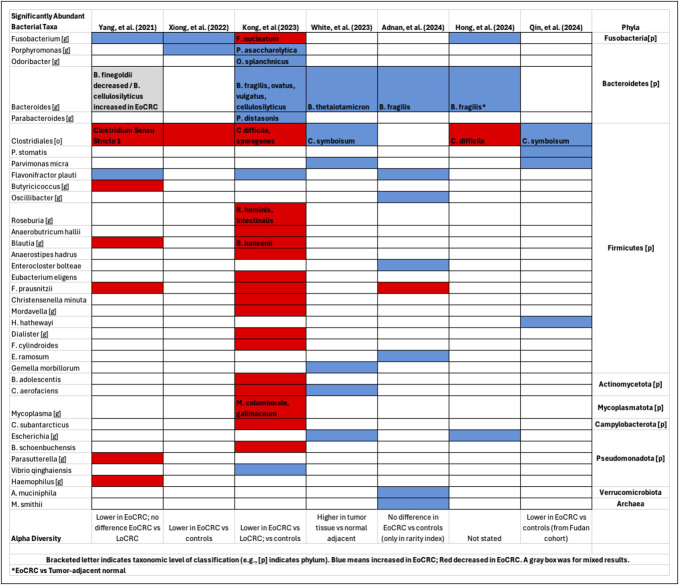
Enteric microbiota alterations between EoCRC and age-matched controls. Reported alterations of the enteric microbiota that featured comparisons of EoCRC vs age-matched controls were summarized. Blue and red boxes, respectively, indicate reported increases and decreases in relative abundance associated with EoCRC or gray if mixed. Bracketed letter indicates taxonomic level of classification (e.g., [p] indicates phylum). When available, species data are included within the genus taxa box. A summary of alpha diversity findings is included at the bottom of the table for each study. Bacterial taxa were grouped based on similar phyla, and there were Archaea noted as well. In the study by Hong et al, the increased abundance of *B. fragilis* in EoCRC was found only in comparisons looking at tumor-adjacent tissue. EoCRC, early-onset colorectal cancer.

Comparing EoCRC with age-matched controls found more consistent differences. *Fusobacterium* again was increased in EoCRC cases (40,87,89), except for 1 study (91), suggestive that EoCRC arises from luminal environments more procarcinogenic than LoCRC, especially compared with those without cancer (Figure 2).

Several genera from the phyla Bacteroidetes were increased in EoCRC, including *Porphyromonas* (40,91) (specifically *P. asaccharolytica* (91)) and *Bacteroides* (84,85,87,89,91). *B. fragilis* was increased in EoCRC in several studies (85,87,91), concordant with prior findings in CRC (47,53) (Figure 2). Enterotoxigenic *B. Fragilis* produces fragilysin that can activate Wnt/B-catenin and nuclear factor kappa B pathways, inducing cell proliferation and inflammation which may trigger carcinogenic/inflammatory cascades in colonocytes to trigger myeloid cell–dependent distal colon tumorigenesis (100,101). *Porphyromonas*, and specifically *P. asaccharolytica* (63), has been well associated with LoCRC (42,43,63,102), another commonality with EoCRC.

Phyla Firmicutes had mixed results. *Clostridioides  difficile* was decreased in EoCRC (87,91), whereas *C. symbiosum* was increased (84,92). *C. symbiosum* requires further investigation into its role in CRC. *C. difficile*, a pathogenic noncommensal associated with LoCRC, being depleted in EoCRC suggests nosocomial and opportunistic infections, and their predisposing risk factors, are less common in younger individuals and may play a smaller role in EoCRC pathogenesis (103).

Other bacteria elevated in EoCRC include *Parvimonas micra* (84,92) and *Flavonifractor plauti* (85,89,91) (Figure 2). Recent work with LoCRC has elucidated associations with oral microbiota, including *Parvimonas micra* now associated with EoCRC (59,63,64,104,105). *Flavonifractor plauti* is inversely associated with flavonoid compound ingestion, possibly linking dietary medicated influences to risk reduction (106).

Bacterial taxa significantly decreased in EoCRC include *Blautia* (89,91) (particularly *B. hansenii* (91)) and *Faecalibacterium prausnitzii* (85,89,91). *Blautia* and *Faecalibacterium* are associated with right-sided CRC, consistent with EoCRC commonly originating in the distal colon (107). *Collinsella aerofaciens* was mixed (84,91). Finally, *Escherichia* was increased in 2 studies (84,87), similar to prior findings with CRC (54) (Figure 2).

Alpha diversity was consistently lower in EoCRC compared with age-matched controls (40,89,91,92). Because the overall trend was more consistent than comparing CRC subtypes, alpha diversity may fare better as a biomarker for EoCRC in certain age populations at risk for cancer rather than classifying CRC subtypes.

**Figure 3.**
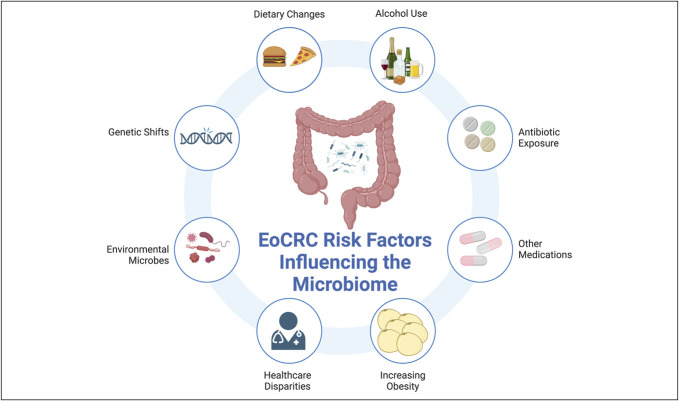
EoCRC risk factors influence the enteric microbiome. Environmental and host factors independently associated with colorectal cancer risk may also influence the gastrointestinal microbiome. These represent potential interlinked associations that could mediate the increased risk of EoCRC in the age cohort populations born after 1960 and presents potential targets for further investigation. EoCRC, early-onset colorectal cancer. Created in BioRender. Luevano J (2025). https://BioRender.com/g32x299.

Unique microbiome signals in EoCRC are promising, but only by improved profiling of structural and functional changes during pathogenesis may we elucidate potential causative factors for the increasing incidence of EoCRC in specific age populations that could become targets for screening or intervention. This influence may be further coupled with the multitude of risk factors well-established with CRC, which may induce their carcinogenic effects through the microbiome (Figure 3).

### Microbiome as noninvasive screening biomarker

Four studies evaluated the enteric microbiome as a biomarker (88,89,91,92). From their Fudan cohort, Yang et al, developed a random forest classifier (RFC) identifying EoCRC vs controls (AUC = 0.8657 on training, AUC = 0.7952 on validation). A new model combining all cases and controls accurately classified EoCRC (AUC = 0.878) and LoCRC (AUC = 0.8667) (89). Subsequent work featuring additional data had more variable accuracy, from an AUC = 0.5629 (Guangzhou cohort) up to 0.9042 (meta-dataset trained, Guangzhou tested) (92). The meta-cohort featured 293 LoCRC and 167 EoCRC Guanzhou cases, 379 LoCRC and 185 EoCRC Fudan cases, and aggregated 72 EoCRC and 528 LoCRC cases from 8 studies. Larger, more diverse cohorts improved the accuracy of microbiome-based prediction tools.

Using a RFC, Kong et al, evaluated the discriminatory ability of multiomic markers (bacterial metagenomics, stool metabolomics, and/or gene expression) for EoCRC vs controls with good accuracy (91). Forty-nine species (AUC = 0.8828 on test dataset, 0.7734 on validation), 36 stool metabolites (0.8828, 0.7535), 59 KO-genes (0.8395, 0.7552), and 27 integrated markers (0.9102, 0.7847) were used as prediction features (91). Jayakrishnan et al, used a Data Integration Analysis for Biomarker Discovery using latent variable approaches for Omics studies (DIABLO) classifier to discriminate EoCRC from LoCRC, featuring 16S data (from cases, AUC = 0.61) and untargeted serum metabolomics (AUC = 0.98). Controls were successfully age stratified through serum metabolomics (AUC = 0.79) (88).

This illustrates that additional multiomic markers improve on present noninvasive prediction methods and highlights the importance of increasing the accuracy of future predictive models by testing and tuning on broader populations. Meta-analyses provide a means to draw further conclusions from available data and can help address lack of diverse representation (81,92). Kharofa et al, used publicly available metagenomic data from 10 studies to identify microbial associations with EoCRC (81). Although the studies did not explicitly subset for EoCRC, in aggregate they included sufficient EoCRC cases to identify significant associations. Qin et al, incorporated EoCRC and age matched controls from a new cohort, prior cohort, and 8 publicly available datasets to develop a predictive pipeline with high accuracy featuring multinational data (92).

The studies reviewed include 847 US and 510 Chinese EoCRC cases; however, they feature methodological differences in sample collection (tissue vs feces) and sequencing methods for data uploaded to public repositories. Overcoming these differences requires careful consideration in statistical analyses. Downstream standardization would allow for evaluation of the effects from differing computational methods. Future studies should aim to sample throughout the gastrointestinal tract (i.e., tissue, saliva, and colon/stool) as well as serum and follow standardized processing pipelines to optimize cross-cohort characterization.

Deeper sequencing coupled with larger cohorts would improve species and strain level characterization in EoCRC to better determine microbial biomarkers. Studies incorporating data from non-EoCRC focused studies can be used but would be limited to those with publicly available metadata to accurately stratify by age and still face similar challenges stemming from methodological differences.

## FUTURE DIRECTIONS AND DISCUSSION

Studies have demonstrated potential pathways by which pathogenic bacteria alter the luminal microenvironment in a way that is conducive to the pathogenesis of CRC (41,46,48,52,54,55,57,108–112). However, CRC carcinogenesis is a multistep process influenced by host, environmental, and microbial factors (Figure 3). One postulated causal factor is antibiotic exposure which has a mild association with EoCRC, but significance was lost after covariate adjustment (113). The true influence of early-life antibiotic use has yet to be characterized through longitudinal studies.

### Microbiome-metabolic associations

**Figure 4.**
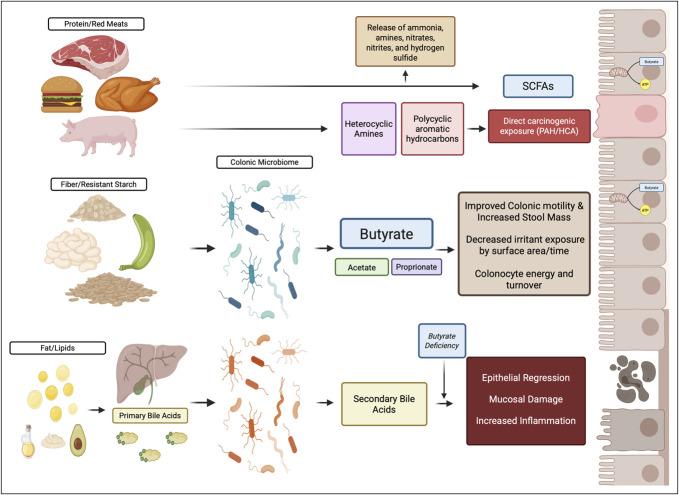
Microbial mediation of dietary factors. Dietary factors that affect CRC risk are highlighted in 3 major food groups (protein/red meat, fiber/resistant starch, and fat/lipids). Although direct microbial metabolism of proteins is not well elucidated, they can lead to the production of SCFAs as well as toxic compounds that influence colon health. Fiber/resistant starches can be metabolized by the microbiome to produce SCFAs, in particular butyrate, that has many beneficial impacts on colonocytes. Fats and lipids induce BA secretion, and these can be biotransformed into secondary BAs that have carcinogenic potential, which is compounded when butyrate is deficient. BA, bile acid; CRC, colorectal cancer; SCFA, short-chain fatty acid. Created in BioRender. Luevano J (2025). https://BioRender.com/mv0u0sr.

Certain host risk factors are mediated via the microbiome through SCFA production and BA biotransformation. Specific bacterial enzymes allow for deconjugation of primary BAs, which promotes retention in the lumen through to the distal colon where other bacteria can use them as substrates, and deconjugated BAs can be further transformed into secondary BAs (94) (Figure 4). Extreme diet shifts can alter the microbiome in just 2 weeks, with measurable microbiome and metabolome alterations that increase saccharolytic fermentation and butyrogenesis while suppressing secondary BA synthesis (114). *Blautia*, found to be decreased in EoCRC (85,89,91), harbors 3α-hydroxysteroid dehydrogenase (HSDH) activity to metabolize BA (115), whereas *B. Fragilis* (85,87,91), increased in EoCRC, can harbor 7α-HSDH activity, demonstrating the nuances in BA metabolism (116). However, further work directly linking these functions to EoCRC risk is needed.

Dietary fiber increases stool mass, bowel transit, and bacterial fermentation of resistant starches into SCFAs, improving colonocyte turnover, and *in vitro* inducing apoptosis in CRC cells (117–121). Increased stool mass and reduced transit time can lower colonocyte exposure to carcinogens through physical dilution and shorter exposure time (Figure 4).

Balanced protein, fat, and fiber intake improves butyrate production reducing CRC risk (50), whereas Western diets promote protein fermentation and BA deconjugation, which is proinflammatory and damaging to colonocytes (51). The carcinogenic ability of secondary BAs are potentiated by butyrate deficiency (50). *Roseburia* and *Faecalibacterium* that produce butyrate have decreased levels in CRC (50) and EoCRC (85,89,91). This is particularly of note as butyrate-resistant colonocytes may retain growth advantages and potentially form more aggressive cancers through altered Histone Deacetylase function and insulin sensitivity/glucose tolerance (122,123).

Although human studies have yet to delineate mechanistically how metabolites from dietary fat influence CRC, animal models found that high-fat diets stimulated BA secretion, causing epithelium regression, mucosal damage, and increasing CRC risk (124,125). Heavy protein intake has been hypothesized to produce carcinogens like heterocyclic amines (HCA) and polycyclic aromatic hydrocarbons during excessive cooking, with HCA being highly mutagenic *in vitro* (126–128). Protein fermentation differs from saccharolytic fermentation in that it produces many similar SCFAs from a carbon skeleton, but also releases potentially toxic nitrogenous and sulfur metabolites such as ammonia, amines, nitrates, nitrites, and hydrogen sulfide (129) (Figure 4).

By harnessing the microbiome, and better characterizing how known CRC risk factors mediate their influence through the microbiome in EoCRC, we may be able to not only predict for those at risk of EoCRC but reduce that risk (Figures 3 and 4).

### Addressing EoCRC in underrepresented populations

The microbiome is influenced by host factors associated with health care disparities and race, often left out of investigative analyses. Racial disparity exists in CRC diagnosis and treatment and is more pronounced in EoCRC. Improved mortality from screening seen in White Americans is not fully realized in minority populations (130). Cumulative CRC survival and disease-specific mortality is worse for Black Americans compared with White patients across all disease stages, and they are up to 40% more likely to be diagnosed with advanced stage IV CRC (18,75–77,131). Compared with White patients, Black patients had higher toxicity from 5-fluorouracil, lower rates of surgical resection, and shorter surveillance follow-up (99). Although race associates with worse outcomes, ultimately it may be an indirect marker of social and environmental risks that require improved comprehension (20,132–135).

**Figure 5.**
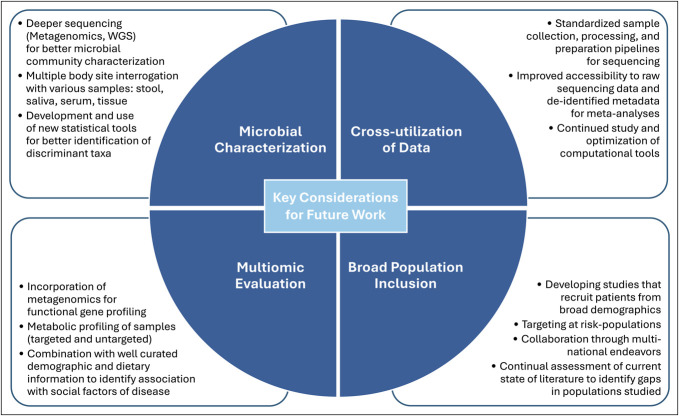
Key considerations to guide future work. Key concepts to improve further characterization of the role of the enteric microbiome in EoCRC and guide future investigative efforts summarized by focus categories. EoCRC, early-onset colorectal cancer.

Pilot studies have investigated racial differences or focused on CRC in Black populations (136–141). Comparing Blacks with other groups found differences that may influence disparities in CRC incidence and outcomes, especially considering differing microbiome baselines associated with host diversity (136,138,142,143). The gap is more pronounced in EoCRC studies. The majority lack racial or ethnic descriptions of their populations (40,78–81,83,85,87,89–92). Three include race descriptions, but feature predominantly White patients (71%–100%) (84,86,88). One abstract discussed differential abundance of taxa by race: with Black patients enriched for *Limosilactobacillus*, *Bacillus*, and *Staphylococcus*, whereas White patients were enriched for *Enterococcus* and *Escherichia-Shigella* (82). The microbiome of EoCRC and LoCRC cases were more similar within Black patients than EoCRC between races; however, population breakdown was not detailed (82). Currently we cannot conclude the influence and importance of race on EoCRC (Figure 5).

Equalizing screening is a successful means to improve health care outcomes. The Delaware Cancer Consortium pioneered a program with navigators for CRC screening, cancer treatment for uninsured, and an emphasis on Black-American cancer reduction. Targeted efforts improved screening for Black patients from 48% in 2001 to 74% 2009, equal to rates in White patients (58%–74%) (144). By the study's end, there was no difference in LoCRC incidence, with mortality improvements lagging but nearly equalizing (144). However, not all health care systems can massively increase colonoscopies. Kaiser Permanente studied noninvasive screening as a method to eliminate disparities and found that proactive annual fecal immunochemical testing and on-request colonoscopy decreased absolute differences between Black and White patients from 21.6 to 1.6 cases per 100,000 (145).

Nonendoscopic screening alternatives based on microbiome and metabolic data demonstrate improved accuracy compared with older serologic markers (e.g., carcinoembryonic antigen and carbohydrate antigen 19-9) (70–72,146–148) or fecal-based immunochemical tests (73,74,149), supporting their diagnostic utility. Recently blood-based cell-free DNA screening was studied in average-risk populations (150), but additional investigation is needed to highlight at-risk and minority groups and build on the demonstrated utility of not only stool metabolite profiling but urine (151,152) and serum (88,152). Future studies should sample various noninvasive sources to better evaluate alterations associated with EoCRC carcinogenesis (Figure 5).

Improved comprehension is required into how demographic differences in vulnerable populations—such as minorities and those with EoCRC—are influencing the enteric microbiome and in turn CRC pathogenesis. Optimally quantifying these influences will require studies that incorporate community level analyses, as well as functional profiling and well-curated demographic information (e.g., dietary and medication information), to develop robust multiomic evaluation. Future tools would benefit from diverse training datasets to optimize development of new predictive tools with consistent accuracy when externally validated (Figure 5).

## CONCLUSION

Our understanding of microbial alterations in patients with EoCRC compared with LoCRC and controls is improving but requires broader sampling in future studies. Evaluation of the microbiome along the gastrointestinal tract, characterization of functional alterations, and inclusion of diverse populations disproportionately burdened with disease will yield further insight into EoCRC. Future studies should capitalize on patient cohorts that serve underrepresented populations to better characterize how their microbiomes influence EoCRC, which may lead to worse outcomes, and develop broadly applicable prediction tools that could in turn be used to expand screening outreach and improve outcomes.

## CONFLICTS OF INTEREST

**Guarantor of the article:** Jesús M. Luévano Jr, MD, MS.

**Specific author contributions:** J.M.L.: conceptualization and analysis of literature, and figure generation and writing-original draft preparation. J.L. and T.S.: supervision and editing. All authors have read and agreed to the published version of the manuscript.

**Financial support:** This work was supported by the American Cancer Society Diversity in Cancer Research Program—Clinician Scientist Development Grant (DICRIDG-21-072-01).

**Potential competing interests:** None to report.

## ABBREVIATIONS:

16S: 16S rRNA sequencing
aoCRC: average-onset colorectal cancer
AUC: area under the curve
BA: bile acid
CA 19-9: carbohydrate antigen 19-9
CEA: carcinoembryonic antigen
CRC: colorectal cancer
DIABLO: Data Integration Analysis for Biomarker Discovery using Latent variable approaches for Omics studies
EoCRC: early-onset colorectal cancer
HCA: heterocyclic amines
HSDH: hydroxysteroid dehydrogenase
LoCRC: late-onset colorectal cancer
NGS: next-generation sequencing
RFC: random forest classifier
RNA-Seq: RNA sequencing
SCFA: short-chain fatty acid
WGS: whole genome sequencing
